# What do young Australian adults know about modifiable risk factors for dementia?

**DOI:** 10.1186/s12889-021-12220-7

**Published:** 2021-11-25

**Authors:** Hannah A. D. Keage, Gabrielle Villani, Amanda D. Hutchinson

**Affiliations:** Justice and Society, University of South Australia, GPO BOX 2741, Adelaide, South Australia 5000 Australia

**Keywords:** Dementia, Knowledge, Risk factors, Public health, Young adults

## Abstract

**Background:**

There are well established modifiable risk factors for late-life dementia. These risk factors account for over 30% of population attributable dementia risk and accrue over the lifespan. Young adults have the greatest potential to reduce their own risk for dementia. This study aimed to investigate what young Australian adults know about dementia and its risk factors, and further, how they estimated these risks.

**Methods:**

An online survey promoted through various social media platforms was completed by 604 young Australian adults aged 18–44 years of age.

**Results:**

Seventy percent of participants had a limited understanding of dementia (identifying cognitive or functional impairment), 25% had a good understanding, with 5% having no understanding. Twenty percent of respondents thought there were no modifiable risk factors for dementia. Less the half of participants agreed with two of the nine established dementia risk factors (hearing loss in midlife and education in early life), with over half of participants agreeing to the remaining seven risk factors. Females consistently judged the risks conferred by the nine established dementia risk factors to be higher than males. Those who were lonely judged the dementia risk conferred by loneliness to be higher than those who were not lonely; and smokers judged the dementia risk conferred by smoking to be less than non-smokers.

**Conclusion:**

Young adults have the greatest potential to change their dementia risk, and these findings show that there are important gaps in knowledge of dementia and its risk factors in this group.

**Supplementary Information:**

The online version contains supplementary material available at 10.1186/s12889-021-12220-7.

## Introduction

Dementia is characterised by progressive cognitive decline and functional impairments [1]. Delaying clinical symptoms and the prevention of dementia are global public health priorities [2, 3]. Despite there being no cure for dementia, there is robust evidence of modifiable risk factors such as hypertension and smoking, which account for 30–50% of cases [2, 4, 5]. Misconceptions and stigma impact the public’s understanding of dementia and their willingness to accept empirically supported risk factor information [6–8]. Common misconceptions include that dementia is a normal part of ageing and that all risk factors are non-modifiable [7, 9–11]. Previous studies have reported that the public’s knowledge of non-modifiable dementia risk factors, such as age and genetic factors, is fair to good, ranging from 50 to 70% [12–16]. However, knowledge of modifiable dementia risk factors for dementia is poor, generally under 40% [11, 13, 14, 16–21].

Empirical knowledge of dementia risk factors needs to be translated into targeted public health strategies and campaigns. The first step is to understand what the public already know about risk factors, how they judge these risks, and what individual differences are associated with these risk judgements. This process is similar to what occurred for skin cancer in the 1980s: knowledge that sun exposure increased the risk of skin cancer, which was conveyed through public health messages such as ‘slip, slop, slap’ in Australia [22]. This campaign began in 1981, with the proportion of people likely to get a suntan decreasing from 61% in 1988 to 35% in 1998 [22].

Livingston and colleagues [4] identified nine modifiable risk factors for dementia across the lifespan and calculated associated population attributable fractions (PAFs). Weighted PAFs accounted for communality, that is, the independent fraction of the dementia population (prevalence) that would be eliminated if that risk factor were eliminated. The study reported that hearing loss in midlife had the highest weighted PAF at 9%, followed by low education in early life at 8%, smoking in late-life at 6%, depression in late-life at 4%, physical inactivity in late-life at 3%, social isolation in late-life at 2%, hypertension in midlife at 2%, diabetes in late-life at 1%, and obesity in midlife at 1% [4]. These were updated recently by Livingston et al. [2], with similar PAFs and the addition of three other modifiable dementia risk factors (alcohol, traumatic brain injury and air pollution).

This study, designed and conducted prior to the 2020 update [2], aimed to explore what young Australian adults know about dementia and its modifiable risk factors, and further, how they judged these risks [4]. Young adults are the most critical segment of the population to investigate in terms of dementia prevention, as these individuals have the greatest potential to modify their risk of developing dementia [2, 4]. This is because young adults can make changes prior to mid- and late-life, when most dementia risk is accrued, and they can establish behavioural habits that are likely to persist throughout adulthood.

## Method

### Participants

The sample included in statistical analyses totalled 604 Australian residents aged 18–44 years of age. Notably, there were 1479 responses, with most excluded responses due to a detected bot, which provided hundreds of identical responses within minutes. Respondents were excluded from participation if they: 1) were under the age 18 and over the age of 44; 2) resided outside of Australia; and 3) had already completed the survey. Based on these criteria, 18 respondents were excluded from participating in the survey. Ethical approval was obtained from the University of South Australia’s Human Ethics Committee (202613), and all methods were performed in accordance with the relevant guidelines and regulations.

### Survey measure

A questionnaire was developed to determine what young adults in Australia know about dementia and the risk factors involved (Supplementary Material) and comprised of 42 questions across three sections and took approximately 10 min to complete (based on [21]). (1) The eligibility section of the survey contained three questions, determining whether individuals met exclusion criteria (e.g. Are you between 18 and 44 years of age?). (2) The demographic section of the survey contained eight questions capturing age, gender, first language, state and postcode currently residing in, as well as previous and current educational qualifications. (3) The dementia content section contained 31 questions that were categorised into seven sub-sections: dementia understanding, knowledge of dementia prevention, knowledge of modifiable risk factors, risk judgement, risk perception, dementia reduction and information sources used. These are detailed below.

#### Dementia understanding

The respondents were asked if they knew what dementia was (forced-choice). Respondents who answered ‘yes’ to this question were then asked to describe their understanding of dementia (open response) and the content was rated by two authors (GV and HADK). The respondents were determined as having a good understanding if they detailed both cognitive and functional impairments or declines [1]; as ‘some understanding’ if they described either cognitive or functional impairments or declines; and as ‘no understanding’ if their response was incorrect or if they responded ‘no’ to the initial question (do you know what dementia is?).

#### Knowledge of dementia prevention

Respondents were asked to rate several different statements regarding dementia on a six-point Likert scale ranging from strongly disagree to strongly agree. These included: Dementia is a normal part of ageing; Dementia is curable; Dementia is preventable (able to be avoided); It is possible to reduce the risk of developing dementia; It is possible to delay the onset of dementia; People my age have a good understanding of dementia.

#### Knowledge of modifiable risk factors

Respondents were asked to rate how likely they believed the nine dementia risk factors detailed in Livingston et al. [4] contributed to developing dementia on a six-point Likert scale (strongly disagree to strongly agree). Each risk factor was assessed separately.

#### Risk judgements

The respondents were asked to estimate the percentage reduction in new cases of dementia if a chosen risk factor in a chosen life stage was eliminated (e.g. estimate the percentage reduction in new cases of dementia if hypertension in mid-life is eliminated). This was presented as a visual analogue scale from 0% reduction to 20% reduction for each of the nine risk factors.

#### Risk factor presence

Respondents were asked whether they had experienced each of the nine dementia risk factors outlined in Livingston et al. [4]. Unsure responses were excluded from analyses. The presence of a risk factor was determined relative to its individual characteristics. The presence of hypertension, hearing loss, depression, obesity, and diabetes was determined if a respondent reported “yes, treated” or “yes, untreated” (i.e. 1 = absent, 2 = present, that is, a higher score conveyed dementia risk). The presence of loneliness (social isolation) was classified if a respondent answered “sometimes” or “always” (i.e. 1 = absent, 2 = present, that is, higher score conveyed dementia risk). The presence of smoking was determined if a respondent answered “currently, occasionally” or “currently, daily”, with previous smoking being classified as not smoking (i.e. 1 = no, 2 = yes, with a higher score conveying dementia risk). Low educational attainment was classified as “finished high school only and not currently studying” or “did not finish high school and not currently studying”, with high educational attainment classified as having completed or currently studying any formal post-school programs (e.g. Bachelor’s degrees or vocational training) (i.e. 1 = low education, 2 = high education, that is, higher score conveys protection from dementia). Physical inactivity was classified as “never” or “sometimes” being physically active (i.e. 1 = physically inactive, 2 = physically active; higher score conveys protection from dementia).

#### Dementia concern and action

Using a drop-box format, the respondents were asked to select their level of concern about developing dementia from five response options ranging from not at all concerned to extremely concerned. Participants were also asked “Will you act to reduce your dementia risk?”, with yes and no response options.

#### Information sources used

Respondents were asked to select from 10 listed information sources any that they believed had influenced their knowledge of dementia. Response options included primary school, secondary school tertiary education, public health campaigns, friends or family members, online, TV and radio, social media, books/magazines, health professionals, other.

### Design

A cross-sectional correlational design was employed. Factors that we assessed descriptively included: dementia understanding, knowledge of dementia prevention, knowledge of modifiable risk factors, risk judgements, risk factor presence, dementia concern and action, along with information sources used. The independent variables included were the presence of individual risk factor presence, dementia understanding, age, and gender. The dependent variables in this study included dementia concern, dementia understanding, risk judgements, and information sources used. Notably, dementia understanding was either an independent variable or a dependent variable, across statistical models.

### Procedure

Data collection occurred over 11 weeks from May 28th to August 13th, 2020. An online survey was developed via REDcap, which could be completed on multiple devices including a desktop, mobile phone, laptop or smart pad. Participants were primarily recruited via advertisements on social media sites (Twitter, Facebook, and Instagram) along with word of mouth. To reduce response biases concerning knowledge of dementia and its risk factors, the survey was introduced as ‘What do you know about ageing?’. Respondents were provided with a participant information sheet at the beginning of the survey and completion and submission of the survey was classified as informed consent. All participants who completed the survey were eligible to enter a gift card draw for one of eight AUD$100 gift cards.

### Statistical analysis

All statistical analyses were conducted in STATA 15.0 [23]; all plots were made in R ggplot [24]. Descriptive statistics are displayed as percentages or means and standard deviations. A series of linear regressions with estimated risk judgements as the outcome, and predictors of age (continuous), gender (male as referent, as compared to female and other), presence of a risk factor (each of the nine outlined by Livingston et al. [4]), and dementia understanding (limited knowledge as referent, as compared to no and good understanding). We ran nine ordinal logistic regressions, with the level of concern about developing dementia as the outcome, and the presence (versus absence) of each dementia risk factor as the predictor. An ordinal logistic regression was also run with dementia understanding as the outcome (none, limited to full understanding) and the total number of information sources as the predictor.

## Results

### Sample characteristics

Table 1 details the demographic characteristics of participants. Participants were predominately female (73%), aged 18–29 years of age (58%) resided in South Australia (56%), spoke English as their first language (91%), and were currently not studying but had completed university (38%).

**Table 1:** Demographic characteristics of respondents (*N* = 604)

		Number	%
Gender	Male	155	25.7
	Female	440	72.8
	Other	9	1.5
Age	18–29	352	58.3
	30–44	252	41.7
State residing	South Australia	336	55.6
	Northern Territory	8	1.3
	Western Australia	50	8.3
	Queensland	33	5.5
	New South Wales	72	11.9
	Victoria	77	12.7
	Australian Capital Territory	7	1.2
	Tasmania	21	3.5
First language	English	550	91.1
	Mandarin	5	0.8
	Arabic	1	0.2
	Vietnamese	6	1.0
	Italian	2	0.3
	Other	40	6.6
Education	Highschool not completed and not studying	9	1.5
	Highschool not completed and studying	7	1.2
	Highschool/TAFE completed and not studying	81	13.4
	Highschool/TAFE completed and studying	130	21.5
	University completed and not studying	229	37.9
	University completed and still studying	148	24.5

### Dementia understanding

5% (*n* = 33) had no understanding of dementia, 70% (*n* = 423) had a limited understanding of dementia (either identifying cognitive impairment/decline or functional impairment/decline), and 25% had a good understanding of dementia (identifying both cognitive and functional impairments/declines).

### Knowledge of dementia prevention

**Fig. 1:**
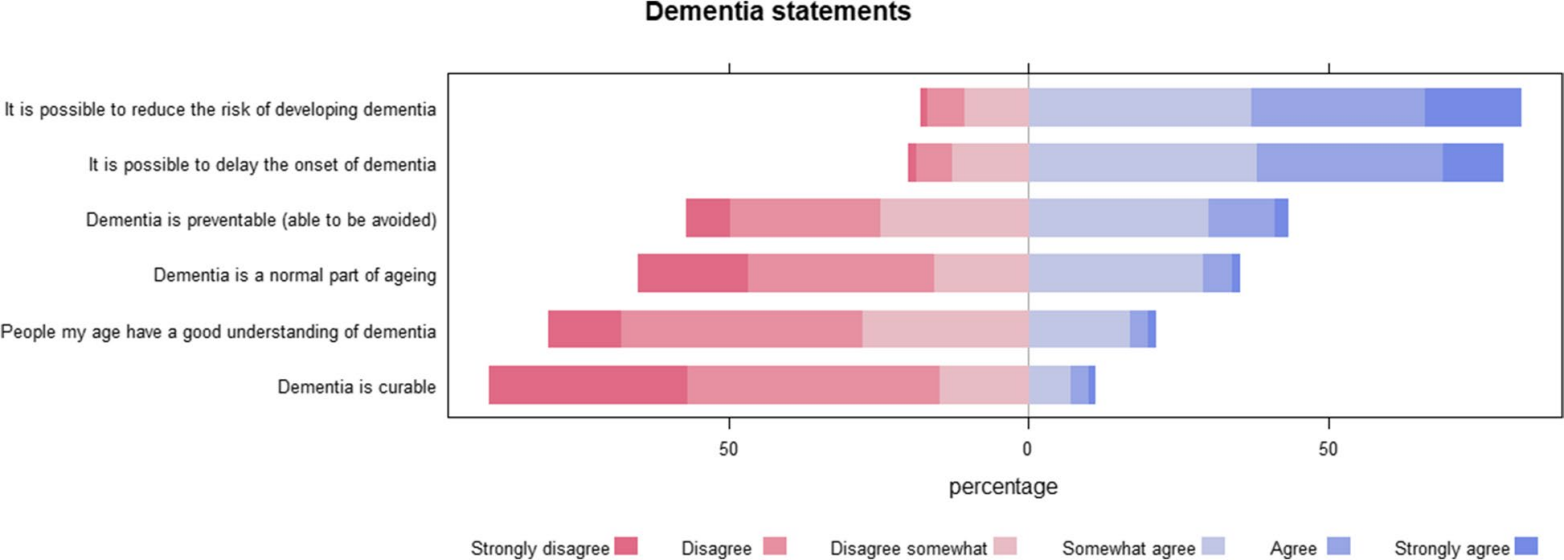
Percentage of respondents agreeing to six key dementia prevention statements

Figure 1 displays the percentage agreement to the six dementia prevention statements. Notably, most people disagreed that dementia is a normal part of ageing; and agreed that it is possible to reduce the risk of developing dementia and that it is possible to delay the onset of dementia.

### Knowledge of modifiable risk factors

**Fig. 2:**
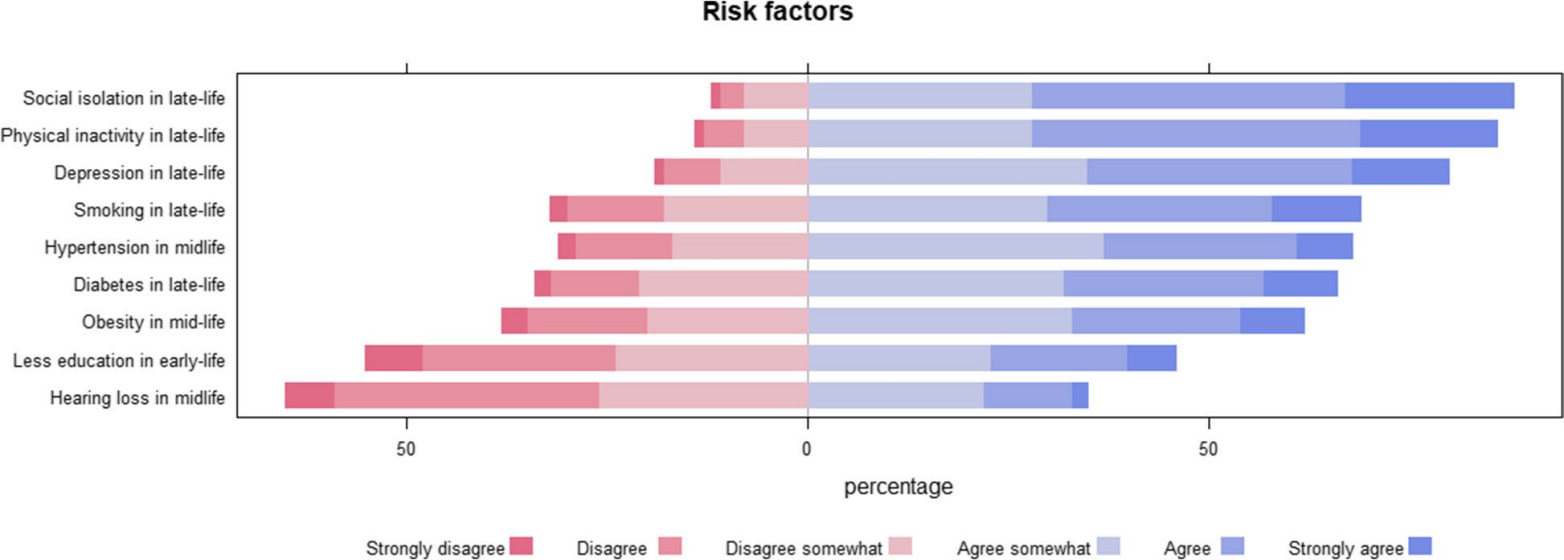
Percentage of respondents agreeing to nine factors known to be dementia risk factors

20% of respondents reported that they did not think there were any modifiable dementia risk factors, and 80% reported that they thought there were modifiable dementia risk factors. Figure 2 displays the percentage agreements relative to the nine known modifiable risk factors. In general, participants agreed to late-life factors more so than early and midlife factors. Less than half of participants agreed that hearing loss in midlife and less education in early-life were risk factors for dementia.

### Risk judgements

Participants were told that each of the nine factors were risk factors and asked to estimate the risks conveyed by each factor (one-by-one) on visual analogue scales of 0 to 20 (e.g. “Estimate the percentage reduction in new cases of dementia if hypertension in midlife is eliminated (between 0 to 20% reduction)”). Table 2 displays the estimated risks conferred by the nine known modifiable dementia risk factors. It can be seen that participants, in general, did not differentiate relative risks between factors, with all being over-estimated as compared to published PAFs from Livingston et al. [4].

**Table 2:** Estimated PAFs (participants estimated these on a visual analogue scale with a minimum of 0 and a maximum of 20%) for the nine dementia risk factors

	Estimated by participants		From Livington et al. [4]
	Mean	SD	PAF
Hypertension in midlife	12.37	4.35	2
Hearing loss in midlife	9.94	4.79	9
Depression in late-life	14.02	4.42	4
Social isolation in late-life	14.32	4.74	2
Less education in early-life	10.41	5.33	8
Obesity in mid-life	12.08	4.74	1
Smoking in late-life	12.75	5.07	6
Physical inactivity in late-life	14.03	4.46	3
Diabetes in late-life	12.29	4.64	1

### Risk factor presence

Nine multiple linear regressions were conducted with the risk judgements as the outcome, and age, gender, dementia understanding and the presence (versus absence) of the risk factor; see Table 3. Females consistently (with most effects also being statistically significant) judged the risks to be greater than males. The presence (i.e. participants’ own history) of a risk factor was not consistently associated with higher risk judgements. Current smokers judged the dementia risk conferred by smoking to be lower than non-smokers; those who reported to be lonely judged the dementia risk conferred by social isolation to be higher than those who were not lonely.

**Table 3:** Results from nine linear regressions, looking at associations between risk judgments and demographic factors along with dementia understanding

		Unstandardised beta	Standard error	*p*	95%CI		Standardised beta	Partial eta
**Hypertension (3% prevalence)**								
Age		0.001	0.0242	0.975	−0.047	0.048	0.001	< 0.001
Gender	Male	–	–	–	–	–	–	0.009
	Female	0.795	0.417	0.057	−0.025	1.614	0.081	
	Other	−1.350	1.679	0.422	−4.647	1.948	−0.034	
Have hypertension (treated or untreated)		0.111	1.103	0.920	−2.055	2.277	0.004	< 0.001
Dementia understanding	None	−0.757	0.837	0.366	−2.401	0.887	−0.038	0.002
	Limited	–	–	–	–	–	–	
	Good	−0.347	0.421	0.410	−1.174	0.480	−0.035	
**Hearing loss (5% prevalence)**								
Age		0.017	0.026	0.515	−0.035	0.069	0.027	0.001
Gender	Male	–	–	–	–	–	–	**0.021**
	**Female**	**1.576**	**0.453**	**0.001**	**0.687**	**2.465**	**0.147**	
	Other	−0.139	1.666	0.934	−3.411	3.133	−0.004	
Have hearing loss (treated or untreated)		0.761	0.920	0.408	−1.046	2.568	0.034	0.001
Dementia understanding	None	0.553	0.871	0.526	−1.158	2.264	0.027	0.001
	Limited	–	–	–	–	–	–	
	Good	0.251	0.463	0.587	−0.657	1.160	0.023	
**Depression (31% prevalence)**								
Age		−0.023	0.025	0.355	−0.071	0.026	−0.038	0.001
Gender	Male	–	–	–	–	–	–	**0.013**
	**Female**	**1.149**	**0.423**	**0.007**	**0.318**	**1.980**	**0.116**	
	Other	1.886	1.533	0.219	−1.126	4.897	0.053	
Have depression (treated or untreated)		0.479	0.397	0.228	−0.301	1.259	0.050	0.003
Dementia understanding	None	−0.448	0.870	0.607	−2.158	1.261	−0.022	0.002
	Limited	–	–	–	–	–	–	
	Good	0.122	0.424	0.774	−0.711	0.954	0.012	
**Social isolation (67% prevalence)**								
Age		0.008	0.026	0.761	−0.043	0.058	0.012	< 0.001
Gender	Male	–	–	–	–	–	–	**0.018**
	**Female**	**1.428**	**0.442**	**0.001**	**0.561**	**2.296**	**0.134**	
	Other	−0.230	1.621	0.887	−3.414	2.953	−0.006	
Experience loneliness (sometimes or always)		**0.809**	**0.409**	**0.048**	**0.006**	**1.612**	**0.080**	**0.007**
Dementia understanding	**None**	**−1.944**	**0.858**	**0.024**	**−3.629**	**−0.258**	**−0.093**	**0.009**
	Limited	–	–	–	–	–	–	
	Good	−0.383	0.448	0.393	−1.262	0.496	−0.035	
**Education (92% prevalence)**								
Age		−0.022	0.029	0.444	−0.079	0.0348	−0.311	0.001
Gender	Male	–	–	–	–	–	–	**0.019**
	**Female**	**1.626**	**0.498**	**0.001**	**0.648**	**2.605**	**0.136**	
	Other	2.900	1.838	0.115	−0.708	6.509	0.066	
> High school education		0.994	0.792	0.210	−0.562	2.550	0.051	0.003
Dementia understanding	None	0.952	0.972	0.328	−0.958	2.862	0.041	0.003
	Limited	–	–	–	–	–	–	
	Good	−0.413	0.510	0.416	−1.408	0.583	−0.033	
**Obesity (15% prevalence)**								
Age		0.352	0.268	0.189	−0.017	0.088	0.055	0.003
Gender	Male	–	–	–	–	–	–	**0.021**
	**Female**	**1.398**	**0.452**	**0.002**	**0.510**	**2.287**	**0.131**	
	Other	−1.784	1.718	0.299	−5.158	1.590	−0.044	
Obese (treated or untreated)		0.408	0.561	0.467	−0.693	1.510	0.031	0.001
Dementia understanding	None	1.313	0.897	0.144	−0.449	3.074	0.061	0.005
	Limited	–	–	–	–	–	–	
	Good	0.434	0.456	0.341	−0.462	1.330	0.040	
**Smoking (14% prevalence)**								
**Age**		**0.070**	**0.028**	**0.012**	**0.015**	**0.124**	**0.103**	**0.011**
Gender	Male	–	–	–	–	–	–	0.006
	Female	0.870	0.474	0.067	−0.062	1.801	0.076	
	Other	1.557	1.752	0.374	−1.884	5.000	0.037	
Current smoker		**−1.233**	**0.601**	**0.041**	**−2.412**	**−0.053**	**−0.086**	**0.007**
Dementia understanding	None	−0.883	0.922	0.339	−2.693	0.928	−0.040	0.005
	Limited	–	–	–	–	–	–	
	Good	0.621	0.480	0.196	−0.322	1.565	0.053	
**Physical inactivity (35% prevalence)**								
**Age**		**0.054**	**0.024**	**0.026**	**0.006**	**0.102**	**0.091**	**0.008**
Gender	Male	–	–	–	–	–	–	0.004
	Female	0.648	0.418	0.121	−0.172	1.468	0.065	
	Other	0.165	1.536	0.915	−2.853	3.182	0.004	
Always physically active		0.298	0.380	0.433	−0.449	1.046	0.032	0.001
Dementia understanding	None	−1.379	0.813	0.091	−2.976	0.219	−0.070	0.006
	Limited	–	–	–	–	–	–	
	Good	0.248	0.425	0.559	−0.586	1.082	0.024	
**Diabetes (2% prevalence)**								
Age		0.345	0.026	0.178	−0.157	0.085	0.055	0.003
Gender	Male	–	–	–	–	–	–	**0.019**
	**Female**	**1.474**	**0.443**	**0.001**	**0.603**	**2.345**	**0.140**	
	Other	0.956	1.701	0.575	−2.386	4.297	0.024	
Diabetes (treated or untreated)		0.356	1.273	0.780	−2.144	2.856	0.011	< 0.001
Dementia understanding	None	−1.132	0.855	0.186	−2.810	0.547	−0.056	0.003
	Limited	–	–	–	–	–	–	
	Good	0.053	0.447	0.906	−0.825	0.931	0.005	

### Dementia concern and action

To the question “How concerned are you about developing dementia?”, the response pattern was: 16% (*n* = 99) not at all concerned, 24% (*n* = 204) slightly concerned, 25% (*n* = 153) somewhat concerned, 18% (*n* = 107) moderately concerned, and 7% (*n* = 41) extremely concerned. Ordinal logistic regressions showed that those with current hearing loss, depression, and loneliness rated their concern for developing dementia to be higher. Notably, age and gender were not significantly related to dementia concern, and their presence in this series of ordinal regressions did not affect results; therefore, we present the unadjusted estimates in Table 4. To the question “Will you act to reduce your dementia risk”, 86% answered yes (i.e. 14% said they would not act).

**Table 4:** Estimates from nine ordinal regressions assessing associations between concern for developing dementia (as a five-level outcome: not concerned at all, slightly concerned, somewhat concerned, moderately concerned, extremely concerned) and the presence (versus absence of each risk factor)

	Unstandardised beta	SE	*p*	95%CI	
Have hypertension (treated or untreated)	0.844	0.436	0.053	−0.010	1.700
**Have hearing loss (treated or untreated)**	**0.696**	**0.342**	**0.042**	**0.027**	**1.366**
**Have depression (treated or untreated)**	**0.522**	**0.161**	**0.001**	**0.206**	**0.838**
**Experience loneliness (sometimes or always)**	**0.308**	**0.156**	**0.048**	**0.002**	**0.613**
>High school education	−0.096	0.265	0.717	−0.616	0.424
Obese (treated or untreated)	0.227	0.206	0.271	−0.177	0.632
Current smoker	−0.339	0.206	0.101	−0.743	0.066
Always physically active	−0.150	0.155	0.335	−0.454	0.155
Diabetes (treated or untreated)	−0.362	0.470	0.440	−1.283	0.558

### Information sources used

On average, 2.90 information sources were used by respondents (SD = 1.62 sources; range 1–10). Information sources included primary school (20), secondary school (107), university (223), public health campaigns (147), family and friends (385), online (237), social media (111), TV and radio (161), books and magazines (100), health professionals (207), and other (53). An ordinal logistic regression showed that more information sources (predictor in model) was associated with higher dementia understanding (as outcome: none, limited to good understanding): unstandardised beta = 0.173 (SE = 0.054), 95%CI 0.068–0.278, *p* = .001.

## Discussion

We show here that young Australians do not consistently recognise evidence-based dementia risk factors and the magnitude of risk they confer. Less than half of participants agreed that hearing loss in midlife and less education in early-life were risk factors for dementia. We did not expect that the public would accurately judge the risk conferred by each risk factor, we were instead interested in factors associated with response patterns (what factors were associated with assessing factors as conferring little or a lot of risk?). However, individual differences hypothesised to associate with risk judgements did not consistently demonstrate relationships. We would have expected those who experience the risk factor to judge the associated dementia risk to be lower [25], however, this was only the case for smoking (with loneliness displaying the opposite pattern). We also expected that greater dementia understanding would be associated with higher dementia risk judgements, which was only the case for loneliness.

### Understanding of dementia and its prevention

Only 25% of respondents demonstrated a good understanding of dementia, in which they accurately described dementia as a disorder that impairs cognitive functioning and an individual’s ability to perform everyday tasks independently. The majority, 70% (423) of respondents demonstrated some understanding of dementia. All but one respondent identified cognitive impairments, specifically memory, but no functional symptoms. While this knowledge is important, it lacks the depth understanding that impairments resulting from dementia reach other cognitive functions beyond memory, and significantly impairs an individual’s ability to perform everyday tasks. The current study revealed that 5% of respondents demonstrated no understanding of dementia. A lack of understanding could have serious implications, including being reluctant to accept empirically supported dementia information and treatment as well as a delay of risk reduction through the modification of lifestyle factors [6–8]. Utilising more information sources to obtain dementia knowledge was associated with better dementia understanding.

Encouragingly, most respondents agreed to the statements “it is possible to reduce the risk of developing dementia” and “it is possible to delay the onset of dementia”; and disagreed to the statement “dementia is a normal part of ageing”. Most respondents however thought that people their age did not have a good understanding of dementia.

### Understanding of modifiable dementia risk factors

Generally, there was better knowledge of dementia risk factors in a recent meta-analysis that included a broader range of ages [19]. Parial et al. [19] reported the following pooled percentages of knowledge of dementia risk factors: inadequate physical activity 43%, smoking of 29%, hypertension 30%, diabetes 33%, obesity 29% [19]. The roles of cardiometabolic risk factors have been previously highlighted as being particularly poorly understood [7], however, we did not see this pattern, rather hearing loss in midlife and early life education were most poorly understood (with less than 50% agreeing they are risk factors). Whether hearing loss (notably, this factor has the highest PAF) and education are poorly understood specifically, or whether it is that these factors appear in early and mid-life, is unknown. This emphasis on late-life could be reflective of misconceptions about normal ageing and being a condition that is only dealt with in the later years of life.

This study was the first to tell respondents what a dementia risk factor was and then had them estimate the risk. There was very little differentiation between the factors, which likely reflects a lack of awareness around their relative impact. We did not expect that the public would accurately estimate the risk conferenced by well-established risk factors, rather the purpose of the estimation was to explore the individual differences associated with risk judgment [25]. A consistent finding was that women generally judged risk conferred by the factors to be higher than men. This is consistent with research demonstrating that men perceive health risks lower than women [26].

According to expectations, smokers reported the dementia risk conferred by smoking to be lower than non-smokers. Smokers have been shown to value immediate rewards (e.g. sense of enjoyment) over those that are delayed or long-term (e.g. health benefits) and predict the onset of smoking related health consequences to occur later than non-smokers [27]. Smokers also underestimate the mortality effects of smoking [28]. Contrary to expectations, those who reported being lonely reported the dementia risk conferred by social isolation to be higher than those who did not report being lonely. Notably, both these effects were small.

### Public health considerations

The views of the current sample are worrying, as there is an obvious lack of knowledge of dementia risk factors in younger Australian adults. Lessons may be learned from cancer prevention; cancer is commonly attributed to fate or genetics despite evidence for lifestyle factors significantly lowering risk. The Cancer Prevention and Control Research Network focused on the implantation of evidence-based approaches to cancer prevention and, using the Science Impact Framework, highlighted the need to move beyond disseminating scientific findings to create awareness, prompt action and effect change [29]. The results of the current study indicate that there is important work to be done to improve awareness of dementia risk factors in the community to reduce dementia risk.

Education is a critical element in contemplating behaviour change, and lack of knowledge is a major barrier to preventative health behaviours in an age group where the greatest gains are to be made. The general public have reported dementia risk to be a matter of “bad luck” [30]. With 30–50% of late-life dementia risk through to be preventable, this understanding needs to change. We need to develop strategies to improve knowledge around dementia prevention and to increase confidence that this is achievable [31]. In a meta-analysis [7], it was reported that nearly half of respondents agreed that dementia was a normal part of ageing; we only saw a rate of 35% (across all three “agree” response categories). We did see that around half of participants disagreed that dementia was preventable, in line with a recent meta-analysis [7]. Generally, people overestimate the importance of the heritability of dementia and underestimate the potential of prevention [32].

In a qualitative study, both fear of developing dementia and the desire to improve dementia knowledge were considered major motivational factors for adopting a healthier lifestyle [33]. Health behaviours are influenced by multiple social, biological and personal factors: personal beliefs, value of the risky behaviour (e.g. enjoyment of a poor diet), barriers to changing risky behaviours, and risk of the outcome (in this case, dementia) [34]. Individuals need to know about dementia and its risk factors in order to change their behaviour to reduce their dementia risk [33]. Specific interventions can then be designed to motivate behaviour change taking into account individual differences as well as the culture and context for specific populations [34].

It is important to note that our sample were highly educated, as is typically found in convenience samples. Only 14.9% of respondents reported leaving education at a high school or TAFE (vocational education) level, 47.2% were currently studying. Given that dementia knowledge and understanding was only moderate, in this well-educated sample, gives additional weight to the need for a public awareness campaign.

### Limitations and conclusion

We may be underestimating the levels of dementia understanding in our sample given participants had to demonstrate this understanding via an open text response, as this method relies heavily on motivation (to write to a reasonable extent). In terms of our survey, the knowledge of dementia prevention statements were presented in their most basic form, and lacked nuance and detail that many dementia researchers would require in order to provide an answer (e.g. the statement “dementia is preventable” is dependent on the percentage of cases and age of the population being referred to). The sample was biased to residents of South Australia, likely as our social media channels have a bias in audience (authors are based at the University of South Australia). All conditions (e.g. hypertension and obesity) were self-reported, which will carry inaccuracies. Further, for power, we had to collapse “treated” and “untreated” response options when classifying the presence of risk factors, which will also add noise to our signals. Larger sample sizes are needed to disentangle differences between treated and untreated groups. There was also a large variability in the presence of risk factors, which meant that we had more power to detect effects for certain risk factors.

We also only presented established risk factors to participants [4]. We did not present factors known to be statistically unrelated such as height. We certainly discussed an option of including “decoy” risk factors, however, wanted to minimise administration time, and expected knowledge to be poor in this cohort (i.e. we did not expect many participants would identify all nine as risk factors). Given 20% of respondents reported that they did not think there were any modifiable dementia risk factors, and the response profiles of agreement to each risk factor (see Fig. 2), we expect this decision did not have a large effect. Concerningly, over 60% of respondents reported being lonely some or all the time. We did ask respondents to disregard the effects of COVID-19 restrictions, but we believe that COVID-19-related isolation played a part in this response pattern.

It has been reported that those over 60 years of age identify dementia as the most important health issue, at a higher rate than those 18–39 years of age (17 v 2%) [35]. We need to have young adults acknowledge dementia as a health priority, as they have considerable potential to impact their dementia risk. There appears a sizeable proportion of young Australian adults who are unconcerned about developing dementia: 16% were not all concerned and 14% said they would not act to reduce their risk (after learning about dementia risk factors as part of this survey). Dementia risk reduction public health campaigns should include all ages, because only targeting older adults may reinforce the misconception that dementia and its prevention is only relevant to late-life. There does not appear enough awareness of the relationship between lifestyle factors across the lifespan and late-life dementia in young Australian adults.

## Supplementary Information

**Additional file 1.**

## Data Availability

Data will be made available on the first author’s GitHub (https://github.com/hannahkeage/dementiaknowledge). If you have any questions about the survey or data, please email the first author (Hannah.Keage@unisa.edu.au).
