# Supplementary material for: What do young Australian adults know about modifiable risk factors for dementia?
Source: BMC Public Health. 2021 Nov 25;21:2166. doi: 10.1186/s12889-021-12220-7 (PMC8616573; doi:10.1186/s12889-021-12220-7)
Supplement: Supplementary file 1 — Additional file 1. [file 12889_2021_12220_MOESM1_ESM.pdf]

What do you know about ageing and dementia?

Are you between 18 to 44 years of age?

◯ Yes
◯ No

Do you currently reside in Australia?	◯ Yes<br>◯ No

Have you completed this survey before?	◯ Yes<br>◯ No

How old are you?

- ◯ 18
- ◯ 19
- ◯ 20
- ◯ 21
- ◯ 22
- ◯ 23
- ◯ 24
- ◯ 25
- ◯ 26
- ◯ 27
- ◯ 28
- ◯ 29
- ◯ 30
- ◯ 31
- ◯ 32
- ◯ 33
- ◯ 34
- ◯ 35
- ◯ 36
- ◯ 37
- ◯ 38
- ◯ 39
- ◯ 40
- ◯ 41
- ◯ 42
- ◯ 43
- ◯ 44

What is your gender?

- ◯ Male
- ◯ Female
- ◯ Other

What is your first language?	◯ English<br>◯ Mandarin<br>◯ Arabic<br>◯ Cantonese<br>◯ Vietnamese<br>◯ Italian<br>◯ Other
Please specify.	__________________________________

What state or territory do you reside in?	◯ SA<br>◯ NT<br>◯ WA<br>◯ QLD<br>◯ NSW<br>◯ VIC<br>◯ ACT<br>◯ TAS<br>◯ Other
Please specify.	__________________________

What postcode do you reside in?

____

Are you currently undertaking any formal education?

◯ Yes
◯ No

What formal education are you currently undertaking?

- ◯ Less than Year 12 or equivalent
- ◯ Year 12 or equivalent
- ◯ Vocational qualification
- ◯ Associate diploma
- ◯ Undergraduate diploma
- ◯ Bachelor degree (including honours)
- ◯ Postgraduate diploma (including graduates)
- ◯ Master's degree
- ◯ Doctorate

What is the highest level of education you have completed?	◯ Less than Year 12 or equivalent<br>◯ Year 12 or equivalent<br>◯ Vocational qualification<br>◯ Associate diploma<br>◯ Undergraduate diploma<br>◯ Bachelor degree (including honours)<br>◯ Postgraduate diploma (including graduates)<br>◯ Master's degree<br>◯ Doctorate

Do you know what dementia is?	◯ Yes<br>◯ No

Please describe your understanding of dementia.

Dementia describes a collection of symptoms that are caused by disorders affecting the brain (Dementia Australia, 2020). Dementia affects thinking, behaviour and the ability to perform everyday tasks. The most common type of dementia is Alzheimer's disease, but there are many others such as Vascular dementia and Frontotemporal dementia. Dementia is most common in late-life, that is, after 65 years of age.

Do you know anyone, or have you known anyone with dementia?

◯ Yes
◯ No

Please indicate their relationship to you.	☐ Partner/spouse<br>☐ Sibling<br>☐ Parent/parent in law<br>☐ Grandparent<br>☐ Aunty or uncle<br>☐ Cousin<br>☐ Friend<br>☐ Neighbour<br>☐ Client<br>☐ Other
Please specify.	__________________________________

How concerned are you about developing dementia?	◯ Not at all concerned<br>◯ Slightly concerned ◯ Somewhat concerned ◯ Moderately concerned<br>◯ Extremely concerned

Indicate the extent to which you agree or disagree with the following statements:

	1. Strongly disagree	2. Disagree	3. Somewhat disagree	4. Somewhat agree	5. Agree	6. Strongly agree
Dementia is a normal part of ageing	○	○	○	○	○	○
Dementia is curable	○	○	○	○	○	○
Dementia is preventable (able to be avoided)	○	○	○	○	○	○
It is possible to reduce the risk of developing dementia	○	○	○	○	○	○
It is possible to delay the onset of dementia	○	○	○	○	○	○
People my age have a good understanding of dementia	○	○	○	○	○	○

What sources have influenced your knowledge of dementia?

- ☐ Primary School
- ☐ Secondary School
- ☐ Tertiary Education
- ☐ Public health campaigns
- ☐ Friends or family members
- ☐ Online
- ☐ TV and radio
- ☐ Social media
- ☐ Books/magazines
- ☐ Health professionals
- ☐ Other

Please specify. ______________________________

A risk factor is something that increases a person's chance of developing a disease or condition. There are two types of risk factors: non-modifiable and modifiable. People have no control over non-modifiable risk factors, and they generally relate to genetics and age. People have control over modifiable risk factors. For example, sun exposure is a modifiable risk factor for developing skin cancer and smoking is a modifiable risk factor for developing lung cancer.

Do you think there are any modifiable risk factors for dementia?

◯ Yes
◯ No

Please list what you think are modifiable risk factors for dementia.

____

There are non-modifiable risk factors for dementia including age that cannot be controlled. There are also modifiable risk factors that can reduce the chance of developing dementia in late-life.

Do you think the following factors are risk factors for developing dementia?

	1. Strongly disagree	2. Disagree	3. Somewhat disagree	4. Somewhat agree	5. Agree	6. Strongly agree
Hypertension (high blood pressure) in mid-life	○	○	○	○	○	○
Hearing loss in mid-life	○	○	○	○	○	○
Depression in late-life	○	○	○	○	○	○
Social isolation in late-life	○	○	○	○	○	○
Less education in early-life	○	○	○	○	○	○
Obesity in mid-life	○	○	○	○	○	○
Smoking in late-life	○	○	○	○	○	○
Physical inactivity in late-life	○	○	○	○	○	○
Diabetes in late-life	○	○	○	○	○	○

Hypertension in mid-life is a risk factor for developing dementia.

Estimate the percentage reduction in new cases of dementia if hypertension in mid-life is eliminated.

0% eliminated — 100% eliminated

(Place a mark on the scale above)

Hearing loss in mid-life is a risk factor for developing dementia.

Estimate the percentage reduction in new cases of dementia if hearing loss in mid-life is eliminated.

0% eliminated — 100% eliminated

(Place a mark on the scale above)

Depression in late-life is a risk factor for developing dementia.

Estimate the percentage reduction in new cases of dementia if depression in late-life is eliminated.

0% eliminated — 100% eliminated

(Place a mark on the scale above)

Social isolation in late-life is a risk factor for developing dementia.

Estimate the percentage reduction in new cases of dementia if social isolation in late-life is eliminated.

0% eliminated ________________ 100% eliminated

(Place a mark on the scale above)

Less education in early-life is a risk factor for developing dementia.

Estimate the percentage reduction in new cases of dementia if less education in early-life is eliminated.

0% eliminated — 100% eliminated

(Place a mark on the scale above)

Obesity in mid-life is a risk factor for developing dementia.

Estimate the percentage reduction in new cases of dementia if obesity in mid-life is eliminated.

0% eliminated — 100% eliminated

(Place a mark on the scale above)

Smoking in late-life is a risk factor for developing dementia.

Estimate the percentage reduction in new cases of dementia if smoking in late-life is eliminated.	0% eliminated ... 100% eliminated<br>(Place a mark on the scale above)

Physical inactivity in late-life is a risk factor for developing dementia.

Estimate the percentage reduction in new cases of dementia if physical inactivity in late-life is eliminated.

0% eliminated 100% eliminated

(Place a mark on the scale above)

Diabetes in late-life is a risk factor for developing dementia.

Estimate the percentage reduction in new cases of dementia if diabetes in late-life is eliminated.

0% eliminated 100% eliminated

(Place a mark on the scale above)

Do you have hypertension?	◯ No ◯ Yes (treated)<br>◯ Yes (untreated) ◯ Unsure
Do you have hearing loss?	◯ No ◯ Yes (treated)<br>◯ Yes (untreated) ◯ Unsure
Have you been diagnosed with depression?	◯ No ◯ Yes (treated)<br>◯ Yes (untreated) ◯ Unsure
Do you have diabetes?	◯ No ◯ Yes (treated)<br>◯ Yes (untreated) ◯ Unsure
Are you obese?	◯ No ◯ Yes (treated)<br>◯ Yes (untreated) ◯ Unsure
Do you smoke?	◯ Never ◯ Previously<br>◯ Sometimes ◯ Always
Besides any isolation in relation to COVID-19, do you ever experience feelings of loneliness?	◯ Never ◯ Sometimes<br>◯ Always
Are you physically active?	◯ Never ◯ Sometimes<br>◯ Always

Now that you are aware of the risk factors for developing dementia, will you act on reducing your risk?	◯ Yes ◯ No

What steps will you take to reduce your risk of developing dementia?

____

At what age do you think people should start to take action to reduce their risk of dementia? (in years) __________________________________

What sources do you think would be the most effective in helping people to understand more about dementia? (e.g. social media) __________________________________
